# Correlation of Anxiety and Depression to the Development of Gastroesophageal Disease in the Younger Population

**DOI:** 10.7759/cureus.32712

**Published:** 2022-12-19

**Authors:** Salomi Paul, Muhammad s Abbas, Sondos T Nassar, Tasniem Tasha, Anjali Desai, Anjana Bajgain, Asna Ali, Chandrani Dutta, Khadija Pasha, Safeera Khan

**Affiliations:** 1 Medicine, California Institute of Behavioral Neurosciences & Psychology, Fairfield, USA; 2 Internal Medicine, California Institute of Behavioral Neurosciences & Psychology, Fairfield, USA

**Keywords:** younger population, asymptomatic erosive esophagitis, psychological factors, non-erosive reflux disease, reflux esophagitis, quality of life, depression, anxiety, gerd, acid reflux

## Abstract

Gastroesophageal reflux disease (GERD) is a condition characterized by the reflux of stomach contents into the esophagus, which leads to heartburn and regurgitation. GERD has been categorized its types according to severity. The categories that have been discussed in this study are reflux esophagitis (RE), non-erosive reflux disease (NERD), and Barrett's esophagus. Our study compared various studies and showed that the subjects with GERD had a high level of anxiety and depression. Gastroesophageal reflux disease has a significant negative impact on the quality of life (QoL) by perturbing daily activities. The majority of GERD patients use antacid drugs to control their acid symptoms. However, these symptoms are sometimes difficult to control, even with the most potent proton-pump inhibitors (PPIs), and these patients tend to have a lower response rate. According to the clinical data, Anxiety and Depression are linked to the development of GERD. A major focus of this study is to explore psychological influences such as anxiety and depression and how they relate to GERD. This study also reviews the effect of these conditions on the younger population. It is concluded that the quality of life (QoL) of subjects with GERD is reduced by depression and anxiety.

## Introduction and background

**Figure 1:**
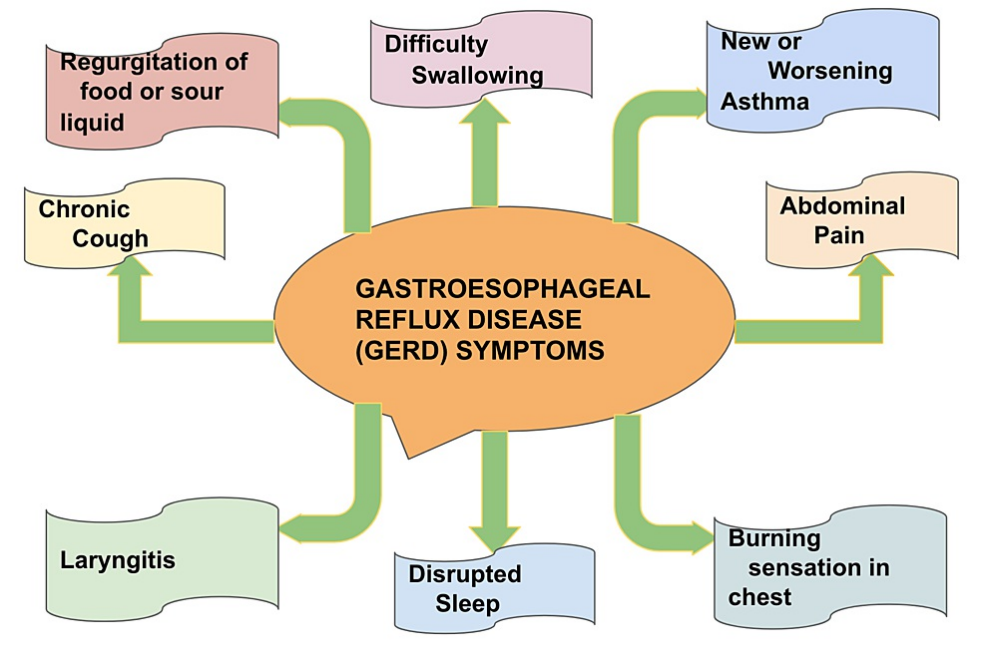
Symptoms of gastroesophageal disease

Gastroesophageal reflux disease (GERD) is a disease that presents with troubling symptoms and complications due to stomach reflux into the esophagus. In developing countries, GERD is one of the most prevalent gastrointestinal disorders [1]. GERD plays a pivotal role in the health-associated quality of life (QOL) by causing difficulties in daily social activities and also in the emotional and physical well-being of the affected patients. The disease can also affect healthy sleep and daily work. Due to the fact that this issue is a digestion disorder, if the lower esophageal sphincter does not close properly, food and liquid can move backward into the esophagus and cause symptoms such as heartburn. The most common complaints related to GERD are heartburn and regurgitation without effort. Weekly, the symptoms of gastroesophageal reflux disease have been reported by 10%-20% of the population [2]. Symptoms of gastroesophageal reflux disease are shown in Figure 1. There is an increase in prevalence worldwide of the two main complications of gastroesophageal reflux disease- Barrett’s esophagus and the adenocarcinoma of the esophagus [2]. *Heliobacter pylori *gastritis, obstructive sleep apnea, obesity, and hiatal hernia are other risk factors. With or without proton pump inhibitors, most patients taking antacids get relief from acid reflux and GERD.

According to Aina and Susman, various medical conditions, such as cardiovascular disease and diabetes, can affect depression [3]. Psychological factors such as depression and anxiety could reduce the threshold for sensation in the human body, and on the other hand, the esophageal stimulation will be increased [3-5]. This study analyzed the symptoms of anxiety and depression and their relation to GERD among the younger population and how this affects the quality of life. 

## Review

According to some studies, physiologic factors such as stress or depression and anxiety may lead to disorders of the gastrointestinal tract [4]. An analysis of cross-sectional data was conducted in different cities in Pakistan. A total of 2,500 people between the ages of 18 and 40 were enrolled in the study. GERD was diagnosed using the Frequency Scale for Symptoms of GERD (FSSG). Symptoms were scored as follows: 0, never; 1, occasionally; 2, sometimes; 3, often; and 4, always. A score of more than 10 indicates GERD. A total of 401 participants were diagnosed with GERD. Anxiety was significantly more common in participants with GERD. Similarly, participants with GERD had a higher prevalence of depression compared to participants without GERD. These studies have found that there is a strong connection between the function of the gastrointestinal system and physiologic factors [4]. These factors will perturb the normal function of the gastrointestinal tract and cause various gastrointestinal disorders including GERD. Based on Jansson's study a 3.2-fold increase in reflux risk was found among subjects with anxiety without depression, 1.7-fold increases in reflux risk were found among subjects with depression without anxiety, and 2.8-fold increases in reflux risk were found among subjects with anxiety and depression [5]. When studies tried to demonstrate the relationship between anxiety and depression and GERD, they delivered contrasting results. Some studies proved they are correlated, whereas the other group refuted the link between anxiety level and acid reflux [4-8]. 

Hence, more studies are needed for the proper understanding of the association between gastroesophageal reflux disease and psychological factors such as anxiety and depression. The younger population had a higher prevalence of the association due to a stressful lifestyle [9]. Many young people lead a stressful life to perform well at their schools, universities, and at jobs, and this stress exacerbates the symptoms of acid reflux [9].

**Figure 2:**
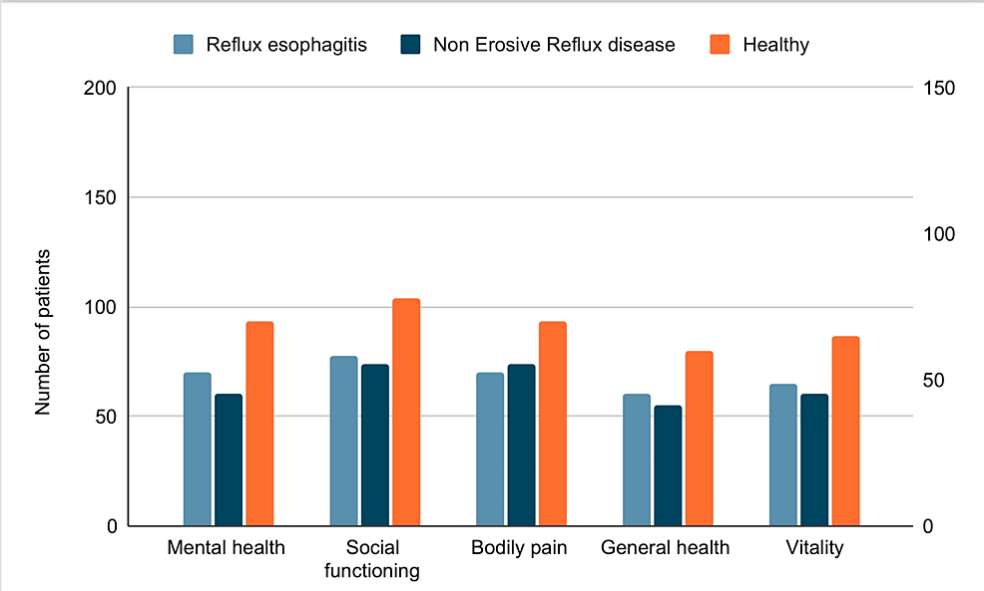
The comparison of the quality of life in three groups: non-erosive reflux disease, reflux esophagitis and healthy individuals. Li et al. study [12].

Li et al. suggest in their study that the psychological range of anxiety and depression was higher in patients with GERD than in normal healthy individuals [10]. Haug et al. found that there is a direct link between psychological disorders and acid reflux [11]. According to Yang et al., non-erosive reflux disease and reflux esophagitis patients had higher anxiety and depression scores than healthy individuals. Their proportions across the three groups have demonstrated moderate or severe anxiety and depression in reflux esophagitis and non-erosive reflux disease. The comparison of quality of life in three groups has been shown in Figure 2 [12].

There are some studies that discovered each factor (anxiety and depression) alone and their influence on gastro-esophageal reflux disease (GERD) development. They also looked at the combined effects that lead to gastroesophageal reflux disease symptoms [12]. A study was conducted to investigate the relationship between these symptoms in the population. A questionnaire was administered to 94,197 residents of Nord-Trøndelag County of Norway (HUNT) about their physical and mental health, demographics, and lifestyle factors. The questionnaires were validly returned by 62,651 people (66.5%). The presence of nausea, heartburn, diarrhoea, and constipation over the past year was self-reported. The Hospital Anxiety and Depression Scale (HADS) was used to assess anxiety disorders and depression. A total of 48% of the population reported at least one of the four gastrointestinal symptoms. A total of 15.3% had an anxiety disorder and 10.4% had a depression disorder based on HADS ratings [11]. In a recent study, Yamasaki et al. compared GERD occurrence in the young population and older population. Their results clearly suggest that the younger population is more exposed to risk factors today compared to 10 years ago. According to their findings, the age more sensitive to risk factors among the younger population is from 30 to 39 years [13].

Bai et al. showed a cross-sectional study conducted in Pakistan. They conducted the study in younger subjects and the age group that enrolled in this study was between 18 to 40 where around 2500 patients were included. Through the Frequency Scale for the Symptoms of gastroesophageal Reflux Disease (FSSG) of the questionnaire, they made the diagnosis of GERD. They assigned a score according to the response of the participants. They use 10 as the minimum standard score for the diagnosis of gastroesophageal reflux disease. A score of Zero = never; One= sometimes; Two= sometimes; Three= often; and Four= always. They later divided the participants into two groups: The Case group (patients who were diagnosed with gastroesophageal reflux disease) and the Testimonial group (patients who were negative for gastroesophageal reflux disease). To assess anxiety and depression the Hospital Anxiety and Depression Scale (HADS) was used. The cut-off score was less than 8 on each sub-scale of anxiety and depression. The demographics of the participants has been shown in Table 1 [4].

**Table 1:** The demographics of the participants and comparing them with positive and negative results of gastroesophageal disease. Bai et al. study [4].

Characteristics	Male	Female	AGE (18-30)	AGE (31-40)	Smoking (Yes)	Smoking (No)	Body Mass Index (Yes)	Body Mass Index (No)
Reflux Disease positive (n=401)	251 (62.5%)	150 (37.4%)	271 (67.5%)	130 (32.5%)	200 (49.8%)	201 (50.2%)	191 (47.6%)	210 (52.4%)
Reflux Disease negative (n=2099)	1,001 (47.6%)	1,098 (52.4%)	974 (46.4%)	1,125 (53.6%)	313 (14.9%)	1,786 (85.1%)	541 (25.7%)	1,558 (74.3%)

The prevalence of anxiety using the HADS score showed 162 (40.3% - less than eight), 139 (59.7% - more than eight) in reflux disease-positive patients, and 411 (19.5% - less than eight), 1,688 (80.5% - more than eight) reflux disease negative patients and the prevalence of depression using the HADS showed 171 (42.6% - less than eight), 230 (57.4% - more than eight) in GERD-positive patients and 385 (18.3% - less than eight), 1,714 (81.7% - more than eight) in GERD-negative patients. Hence, anxiety was significantly more common in participants with GERD (40.3% vs. 19.5%; p<0.01). Similarly, participants with GERD had a higher prevalence of depression compared to participants without GERD (42.6% vs. 18.3%; p<0.01) [4].

For the development of gastroesophageal reflux disease, anxiety may play an important role and to worsen the symptoms of acid reflux even though researchers are not clear on how. Some researchers believe that both pain disorders and gastrointestinal disorders have been related to the brain chemical cholecystokinin (CCK) and may play a huge role in the development of GERD in patients with anxiety disorders. There are various theories that prove anxiety increases stomach acid, slows digestion, and leads to increased muscle tension that puts pressure on the stomach [14-18].

**Figure 3:**
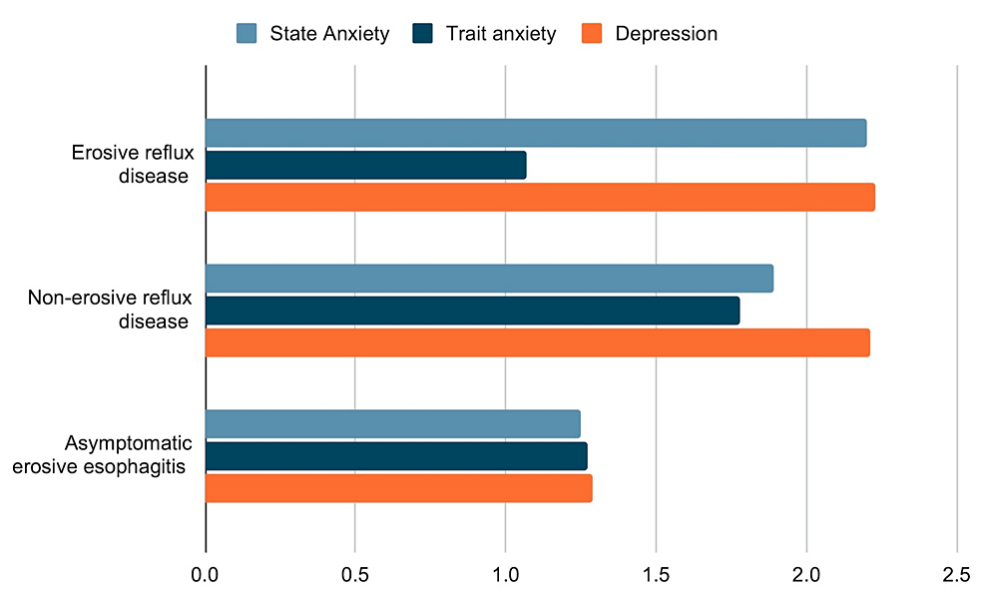
Baseline characteristics of subgroups of gastrointestinal disease and their relationship to anxiety and depression. Choi et al. study [18].

Another study was done in Korea at the Seoul National University Hospital Healthcare System, Gangnam Center from January 2008 to December 2011 based on the general population was a retrospective, cross-sectional study that included 1574 (8.2%) participants that are dealing with reflux symptoms. They recruited young, middle-aged, and elderly subjects for the study. All the participants in their study underwent esophagogastroduodenoscopy, and these subjects were separated into non-erosive reflux disease (NERD), erosive reflux disease (ERD), and asymptomatic erosive esophagitis (AEE), and controls. State-Trait Anxiety Inventory and Beck Depression Inventory were used for anxiety and depression. Their study showed that the subjects with GERD had a high level of anxiety and depression [18]. Baseline characteristics of subgroups of gastrointestinal disease and their relationship to anxiety and depression are shown in Figure 3.

**Figure 4:**
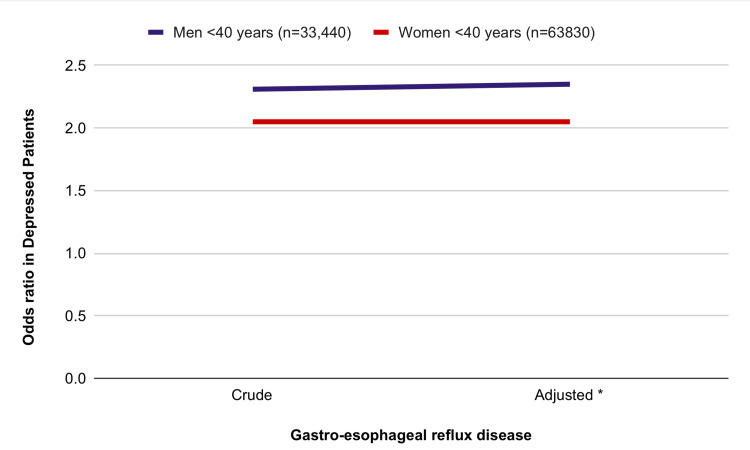
Subgroup analysis of crude and adjusted subjects for gastroesophageal reflux disease in patients with depression stratified according to age and sex. Kim et al. study [19]. *Model adjusted for age, sex, income, region of residence, and histories of hypertension, diabetes, dyslipidemia, ischemic heart disease, and cerebral stroke.

A bidirectional link study of gastroesophageal reflux disease and depression was done by Kim et al. They did the study by bringing out two different nested case-control studies using a national sample report. They studied 19,454 young subjects who are less than 40 years with depression and 34, 698 subjects (<40 years) with gastroesophageal reflux disease. The graph below shows the association between acid reflux and depression among the core and adjustment groups [19]. Subgroup analysis of crude and adjusted subjects for gastroesophageal reflux disease in patients with depression stratified according to age and sex has been shown in Figure 4.

According to Kim et al., there is a prevalence of depression in the United States at roughly 7.6%, the United Kingdom has 11%, in Korea at 6.7%, and is increasing by 0.2% annually [19]. Dr. Martin-merino's study provides further evidence for the link between the presence of reflux symptoms and the use of tricyclic antidepressants (TCAs), anxiolytic and antipsychotic drugs. These factors should be taken into account when managing depression and GERD in primary care [20]. Also, other factors such as obesity, eating habits such as including fried foods, smoking and alcohol consumption, and lack of sleep lead to high stress which may contribute to depression and gastroesophageal reflux disease development [20-23].

There are a variety of treatments that follow the onset of gastroesophageal reflux disease symptoms. It is more important to consider both reflux symptoms and anxiety in the treatment plan. The reason behind this issue is that symptoms like acid reflux have worsened after using them for anxiety. Some medications to avoid in treating anxiety and acid reflux are tricyclic antidepressants, benzodiazepines, and selective serotonin re-uptake inhibitors (SSRIs). Tricyclic antidepressants have proven to reduce the pressure of the lower esophageal sphincter, motility of the esophagus will be impaired due to SSRIs use and later leads to acid reflux episodes., whereas benzodiazepines increase the patient's sensitivity and lead to the painful perception of reflux symptoms by lowering the body's pain threshold mechanism. However, serotonin, serotonin-norepinephrine re-uptake inhibitors (SNRIs) are other medication that is commonly used to treat anxiety. This medication has not been shown to worsen gastroesophageal reflux disease symptoms [18].

## Conclusions

There is an association between gastro-esophageal disease (GERD) and anxiety and depression. Predominantly, GERD remains a disease of the younger population as compared to the middle or old age. Speculation that leads to these findings is poor dietary habits, an unhealthy rigorous lifestyle, lack of sleep, stressful life, etc., which makes the younger population high-risk criteria for GERD. This review revealed that subjects with gastroesophageal reflux disease have higher levels of anxiety and depression. When compared with various pieces of research, it is clear that conditions such as acid reflux, anxiety, and depression play a negative impact on the quality of life of these individuals. Our study suggests that physicians should be more aware of the fact that the range of younger populations exposed to gastroesophageal reflux disease has been increasing tremendously. A multidisciplinary approach to assessing and managing these psychological factors is needed for the treatment of gastroesophageal reflux disease.
